# Human PDE4D isoform composition is deregulated in primary prostate cancer and indicative for disease progression and development of distant metastases

**DOI:** 10.18632/oncotarget.12204

**Published:** 2016-09-23

**Authors:** René Böttcher, Kalyan Dulla, Dianne van Strijp, Natasja Dits, Esther I. Verhoef, George S. Baillie, Geert J.L.H. van Leenders, Miles D. Houslay, Guido Jenster, Ralf Hoffmann

**Affiliations:** ^1^ Department of Urology, Erasmus Medical Center, Rotterdam, The Netherlands; ^2^ Department of Bioinformatics, Technical University of Applied Sciences Wildau, Wildau, Germany; ^3^ Department of Oncology Solutions and Precision Diagnostics, Philips Research Europe, Eindhoven, The Netherlands; ^4^ Institute of Cardiovascular and Medical Science, University of Glasgow, Glasgow, Scotland, UK; ^5^ Department of Pathology, Erasmus Medical Center, Rotterdam, The Netherlands; ^6^ Institute of Pharmaceutical Science, King's College London, London, UK

**Keywords:** phosphodiesterase, cAMP, biomarker, ERG, androgen receptor

## Abstract

Phosphodiesterase 4D7 was recently shown to be specifically over-expressed in localized prostate cancer, raising the question as to which regulatory mechanisms are involved and whether other isoforms of this gene family (*PDE4D*) are affected under the same conditions.

We investigated PDE4D isoform composition in prostatic tissues using a total of seven independent expression datasets and also included data on DNA methylation, copy number and AR and ERG binding in PDE4D promoters to gain insight into their effect on PDE4D transcription.

We show that expression of PDE4D isoforms is consistently altered in primary human prostate cancer compared to benign tissue, with PDE4D7 being up-regulated while PDE4D5 and PDE4D9 are down-regulated. Disease progression is marked by an overall down-regulation of long PDE4D isoforms, while short isoforms (PDE4D1/2) appear to be relatively unaffected. While these alterations seem to be independent of copy number alterations in the PDE4D locus and driven by AR and ERG binding, we also observed increased DNA methylation in the promoter region of PDE4D5, indicating a long lasting alteration of the isoform composition in prostate cancer tissues.

We propose two independent metrics that may serve as diagnostic and prognostic markers for prostate disease: (*PDE*4*D*7 - *PDE*4*D*5) provides an effective means for distinguishing PCa from normal adjacent prostate, whereas *PDE*4*D*1/2 - (*PDE*4*D*5 + *PDE*4*D*7 + *PDE*4*D*9) offers strong prognostic potential to detect aggressive forms of PCa and is associated with metastasis free survival. Overall, our findings highlight the relevance of PDE4D as prostate cancer biomarker and potential drug target.

## INTRODUCTION

With an estimated 417,000 new cases in 2014 in Europe, prostate cancer (PCa) remains the most often diagnosed gender-specific carcinoma for men [1]. The current routine of diagnosing PCa results in a significant number of unnecessary biopsies and treatments of non-cancerous, benign prostate conditions and non-aggressive cancers, leading to severe negative effects for both men and healthcare systems [2, 3].

Next to well-studied pathways such as androgen receptor (AR) and PI3K/AKT, cyclic AMP (cAMP) has been shown to play a role in the development and progression of PCa [4]. The metabolism of cAMP in cells is complex and tailored by spatial and signalling cross-talk considerations involving both a large family of adenylyl cyclases responsible for its synthesis, and a large family of cyclic nucleotide phosphodiesterases (PDEs) responsible for its degradation [5]. It is now well recognized that when particular cAMP degrading PDEs are recruited to specific signalling complexes they create and control cAMP gradients around them, allowing spatially compartmentalised and time-dependent regulation of localized cAMP signalling [6, 7]. Protein domains involved in subcellular localization as well as independent regulatory mechanisms play a pivotal role in these processes, granting PDE isoforms the ability to fulfil functionally independent and unique roles in the cell [6, 8]. Thus, changes in the expression of distinct PDE isoforms can be expected to reprogram downstream signalling pathways during disease development and progression, providing potential targets for novel markers and therapeutic interventions [6]. Indeed, cAMP-degrading PDEs have been associated with several diseases in recent years, including stroke, acrodysostosis and COPD [9–14], and more recently, expression of a specific PDE4D isoform (PDE4D7) has been related to prostate cancer [15, 16].

The PDE4D7 transcript comprises the open reading frame for a long PDE4D isoform that contains both the UCR1 and UCR2 regulatory domains [17]. These protein domains are common to all long PDE4D isoforms with UCR1 being phosphorylated by PKA (cAMP dependent protein kinase A), when cAMP levels within the cell are elevated, leading to enzyme activation [18, 19]. Indeed, activation of long PDE4 isoforms, such as PDE4D7, by PKA provides a fundamental part of the cellular desensitization process to cAMP [6]. Long PDE4 isoforms can also be dynamically regulated through phosphorylation by other key signalling system kinases, namely, by ERK [20], MK2 [21], Cdk5 [22] and AMPK [23]. Additionally, PDE4D7 has been shown [15] to be specifically targeted to the sub-plasma membrane compartment in prostate cancer cells where it regulates local cAMP levels that are linked to cell proliferation [15].

We have previously shown that PDE4D7 is specifically overexpressed in both androgen sensitive PCa cells and in samples from patients with early androgen sensitive prostate disease [15, 16]. However, in marked contrast to this, once PCa cells become androgen insensitive/independent (castration resistant), expression of PDE4D7 declines [15, 16].

Here, we show that PDE4D isoform composition is altered in localized prostate cancer and that it can be used both as a diagnostic as well as a prognostic biomarker. In conjunction with our previous studies, we see that the long transcript isoform PDE4D7 is up-regulated in localized disease compared to normal adjacent prostate (NAP), while its expression diminishes with tumour progression. In contrast to PDE4D7, two other long isoforms, PDE4D5 and PDE4D9, do not undergo an initial up-regulation in primary PCa and instead are increasingly down-regulated during disease progression. Moreover, we suggest that this change in isoform composition may be influenced by the DNA methylation of specific regulatory elements of the PDE4D locus. These findings highlight the potential of using condition-specific mRNA isoforms of the PDE4D gene as biomarkers and potential novel therapy targets to restore benign conditions.

## RESULTS

### The long isoforms PDE4D5 and PDE4D9 are significantly down-regulated in primary prostate cancer, independent of copy number alterations in the PDE4D gene locus

**Figure 1:**
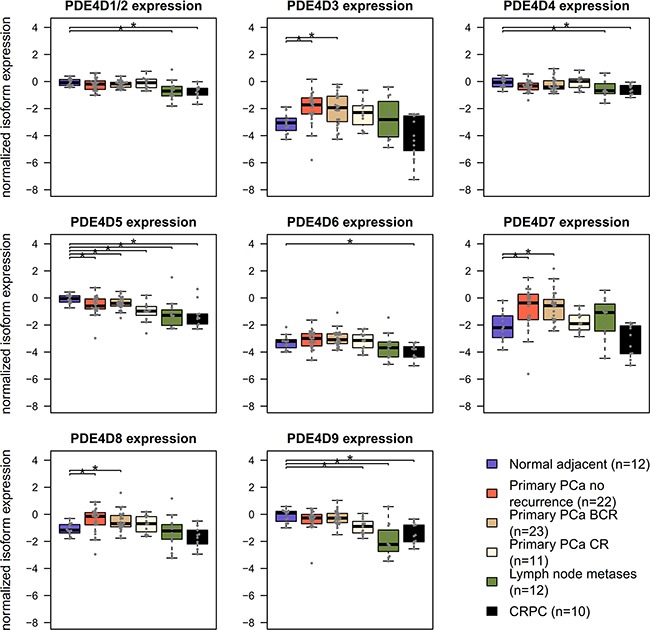
Overview of PDE4D isoform expression in prostatic tissues Normalized PDE4D isoform expression in the EMC dataset across different prostate conditions. CR – clinical recurrence, BCR – biochemical recurrence, CPRC – castration resistant prostate cancer. Significant differences (p < 0.05, Wilcoxon-Mann-Whitney test) are indicated with *.

After previously identifying PDE4D7 as a novel biomarker candidate [16], we wanted to investigate the behaviour of other PDE4D transcript isoforms in PCa development and progression. Therefore, we focused on the nine major human PDE4D isoforms described in RefSeq and conducted a meta-analysis of six publicly available patient cohorts. Our analysis revealed that many PDE4D isoforms are seemingly expressed at stable levels when using Exon Arrays, whereas only PDE4D1/2, PDE4D5, PDE4D7, and PDE4D9 were detectable at higher levels in our independent qRT-PCR cohort of prostate tissues (see Figure 1 and Supplementary Figures S1-S5). These findings were supported by the TCGA PRAD RNA-seq cohort, which mostly agreed with RT-PCR results, despite few outlier samples showing expression of other isoforms (Supplementary Figure S6). Based on these findings, we focused on the above mentioned PDE4D isoforms, as they showed consistent expression profiles in all used cohorts. Using these criteria, we found that both PDE4D5 and PDE4D9 are significantly down-regulated in primary localized PCa when compared to benign samples. Moreover, patient samples derived from castration-resistant prostate cancer (CRPC) showed further down-regulation of both isoforms, in line with our previous findings for PDE4D7 [16]. Likewise, PCa metastasis samples followed this trend, but often displayed higher variance in PDE4D isoform expression, as can be expected given their very heterogeneous genomic background [24].

**Figure 2:**
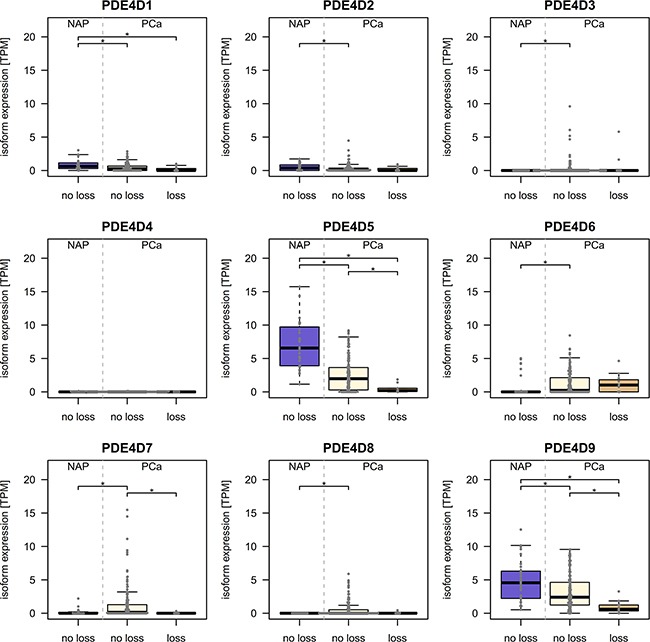
Relation of copy number events and PDE4D expression in the TCGA cohort 32 normal adjacent prostate samples are compared to PCa samples with (n=12) and without (n=171) loss of genetic material in the PDE4D locus to investigate whether decreased expression occurs independently of PDE4D deletions. Significant differences in expression are denoted with * (p < 0.05, Wilcoxon-Mann-Whitney test).

Since, partial or complete deletions of one or both alleles of the *PDE4D* gene have been reported previously in prostate cancer [25–27] we utilized TCGA SNP array data of matching patient samples to assess the potential impact of deletions occurring in *PDE4D* on isoform expression. Although we did observe a significant reduction in gene expression upon loss of genetic material, both isoforms were also expressed at significantly lower levels in PCa samples which did not harbour a deletion when comparing to matching normal samples (Figure 2).

### Androgen receptor and ERG are implicated in transcriptional regulation of PDE4D

Our previous work suggested an association between PDE4D7 expression and the presence of the TMPRSS2-ERG fusion gene [16]. We therefore set out to investigate whether there was any comparable ERG involvement in the expression of PDE4D5, PDE4D7 and PDE4D9 in prostate disease. In order to do this, we assigned localized PCa samples to one of two groups based on an unsupervised clustering of ERG expression values by Partitioning Around Medoids and used available ERG IHC information of the EMC cohort to confirm the validity of this approach. Clustering based grouping showed good concordance with IHC results, assigning four additional samples (10.2%) to the ERG positive group (Supplementary Figure S7).

**Figure 3:**
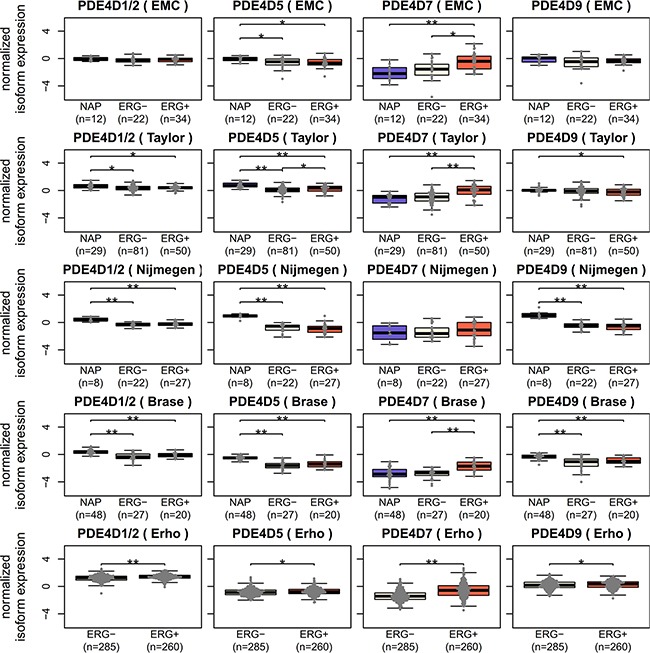
Investigating potential ERG regulation of PDE4D isoforms Since only PDE4D7 has been previously reported as up-regulated in ERG positive PCa samples [16], expression of PDE4D1/2, PDE4D5, PDE4D7 and PDE4D9 was tested in ERG negative and ERG positive samples across five Exon Array datasets (* = p < 0.05, ** = p < 0.001, Wilcoxon-Mann-Whitney test).

Interestingly, while we were able to confirm PDE4D7 overexpression in ERG positive PCa samples, PDE4D1/2 and PDE4D9 seemed unaffected by ERG, whereas PDE4D5 expression was altered significantly in two out of five datasets, suggesting that any connection between PDE4D5 and ERG is weak at best (see Figure 3). Of note, the Erho dataset consistently showed significant changes for all isoforms, however, these likely do not reflect real events, as absolute log2 fold changes were small (|log2FC| < 1) except for PDE4D7 (data not shown). Therefore, ERG linkage discriminates between PDE4D7 and the grouping of PDE4D1/2, PDE4D5 and PDE4D9, where we see differences between these two groups in the change of their expression in prostate disease.

**Figure 4:**
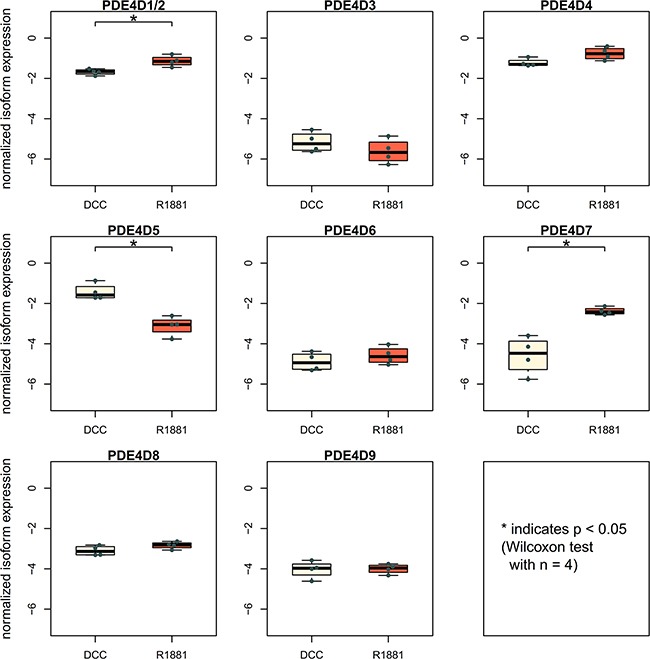
Investigation of androgen receptor involvement in PDE4D expression Expression of PDE4D isoforms in LNCaP cells with or without addition of the synthetic androgen R1881 [28].

To investigate androgen-dependence of PDE4D isoform expression, we incorporated a public dataset of LNCaP cells measured after being kept either in androgen stripped medium (using dextran-coated charcoal - DCC) or after addition of the synthetic androgen R1881 [28]. While PDE4D9 expression was not altered after treatment, both PDE4D5 and PDE4D7 showed significant differences in expression after R1881 addition (Figure 4). Specifically, PDE4D5 expression appeared to be inhibited upon AR stimulation, while PDE4D7 was up-regulated in DCC by the synthetic androgen R1881 in LNCaP cells.

**Figure 5:**
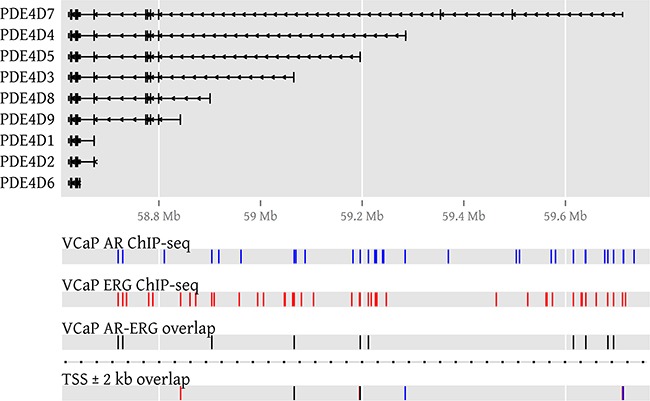
AR and ERG binding peaks in PDE4D in the VCaP cell line To visualize AR and ERG binding in PDE4D, genomic locations of ChIP-seq peaks (GSE14092) denoting AR binding sites are coloured blue, while ERG peaks are coloured in red. If peaks of both transcription factors overlap, the affected genomic regions are coloured in black. A genomic region surrounding each transcription start site (TSS) is used to highlight binding events that could influence transcription.

Next, we made use of public ChIP-seq data from the VCaP PCa cell line [29] treated with R1881 in order to gather further evidence of AR involvement in PDE4D expression. In ChIP-seq, DNA binding proteins and associated chromatin are cross-linked, followed by immunoprecipitation of a protein of interest and subsequent sequencing of the associated DNA fragments, allowing a genome-wide localisation of its DNA binding sites. Overall, we found 31 ChIP-seq peaks for AR in PDE4D, two of which were near the first exon of PDE4D7 (~2 kb and 3 kb upstream), while another was partially overlapping the first exon of PDE4D5 (see Supplementary Table S1). No peaks could be found in proximity to the PDE4D9 transcription start site (TSS), as the closest upstream and downstream peaks were found at an approximate distance of 85.5 kb and 44.2 kb, respectively. Since VCaP harbours the TMPRSS2-ERG gene fusion and ChIP-seq data for ERG was available from the same source, we included it in our analysis and found 43 ERG peaks in the PDE4D gene locus, of which some were found to partially overlap the first exon of each of the long isoforms PDE4D5, PDE4D7 and PDE4D9 (see Figure 5 and Supplementary Table S2). Since the number of ChIP-seq peaks located in PDE4D appears to be rather high, we were wondering whether binding of AR and/or ERG within the gene locus occurs more often as compared to other regions. For this reason, we counted the number of AR and ERG peaks in 21,209 RefSeq gene loci and used these counts to construct empirical cumulative distribution functions (ECDFs) for both transcription factors. These ECDFs model the background distribution of the counts for both AR and ERG across all genes and enable us to calculate in which percentile the peak counts for AR and ERG in PDE4D are falling. Surprisingly, both AR and ERG were among the top 99.9% of all genes (99.953^th^ and 99.995^th^ percentiles, respectively), suggesting a very strong enrichment in AR and ERG binding within the PDE4D gene locus (see Supplementary Figure S8a). However, since PDE4D is a comparably large gene and spans approximately 1.5 Mb of genomic space, we repeated this analysis using more than three million randomly sampled genomic regions of 1.5 Mb size across all major chromosomes. Again, we found that PDE4D was highly enriched in AR and ERG binding peaks (95.151^th^ and 87.624^th^ percentiles, respectively) compared to random genomic stretches of comparable size (Supplementary Figure S8b). As a whole, these data support the observed expression profiles and suggest an involvement of both AR and ERG in overall PDE4D isoform regulation.

### DNA methylation of defined regions in PDE4D is altered in prostate cancer

To further study transcriptional regulation of the *PDE4D* locus, we obtained public data of DNA methylation in PCa patients from Gene Expression Omnibus (GEO) and TCGA and performed statistical analyses to identify hyper- and hypo-methylated regions in PCa as compared to normal adjacent prostate (NAP). The results of three different platforms determining DNA methylation patterns consistently detected hyper-methylated regions, indicating active silencing of several PDE4D promoters in PCa, involving the transcription start site (TSS) of a total of five PDE4D isoforms, namely the short PDE4D1/2 isoforms and the long PDE4D4, PDE4D5 and PDE4D8 isoforms (see Supplementary Figure S9).

To estimate the impact of these differentially methylated regions (DMRs) on isoform expression, we used Affymetrix Human Exon Array samples obtained from the same patients as the MeDIP-seq cohort [30, 31] and calculated Spearman's correlation coefficient for each of the differentially methylated regions (DMRs) and the associated PDE4D isoform. Of the five TSS involved, PDE4D5 showed the strongest negative association (r = −0.571, Supplementary Table S3), while the four DMRs near the PDE4D4 TSS showed varying agreement between methylation and expression measurements, ranging from r = −0.215 to r = −0.394. These results follow the expected behaviour, as increased DNA methylation impedes transcription [32]. Since the PDE4D1 and PDE4D2 expression could not be independently measured with the Exon Arrays, a negative correlation (r = −0.517) was found for both. Lastly, PDE4D8 expression did not show any association with DNA methylation (r = −0.233), agreeing with our observation that this isoform is not consistently expressed in prostate tissues (see Supplementary Figure S1).

### PDE4D isoforms can be used as diagnostic and prognostic signature for prostate cancer: application to prostate biopsies

Since PDE4D7 and PDE4D5 show opposing behaviours in prostatic tissues, we created a diagnostic signature based on the expression of PDE4D7 relative to that of PDE4D5 expression (*PDE*4*D*7 - *PDE*4*D*5). In order to evaluate its performance in distinguishing PCa and non-PCa samples, we carried out ROC analyses in all compatible datasets and compared the resulting AUCs with PCA3 (Supplementary Table S4). Overall, our diagnostic signature performed on par with PCA3, with AUCs ranging from 0.839 to 0.934 compared to 0.857 to 0.921.

**Figure 6:**
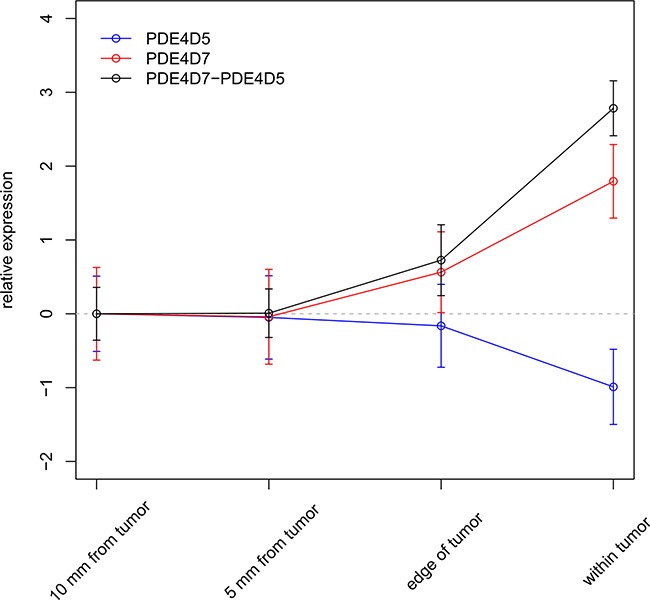
Applying the diagnostic PDE4D signature in needle biopsies Expression of PDE4D5 and PDE4D7 in relation to distance to the tumour as measured by qRT-PCR in prostate tumour biopsies (n = 18). Error bars represent standard error of the mean.

In order to evaluate the value of PDE4D as a clinical biomarker, we used surgical resection materials of eighteen patients and subjected them to needle biopsies to obtain material from distinct areas, simulating both true positive and false negative biopsies (see Supplementary Table S5). In total, four biopsies with gradually increasing distance from the tumour were taken per patient (within tumour, edge of tumour, 5 mm from edge, and 10 mm from edge) and PDE4D5 as well as PDE4D7 expression were measured by qPCR. Ct values of both isoforms were normalized to several reference genes (see Methods) and adjusted to baseline expression in NAP tissue (10 mm from edge). Both expression profiles showed inverse correlation, with PDE4D5 expression decreasing in the vicinity of the tumour, while PDE4D7 expression as well as the diagnostic signature gradually increasing (see Figure 6), confirming our earlier findings. Additionally, a transient change of PDE4D isoform expression at the tumour edge might suggest that nearby adjacent normal tissue is influenced by tumour presence through a ‘field effect’, but could also be due to averaging signals from normal and cancerous cells.

**Figure 7:**
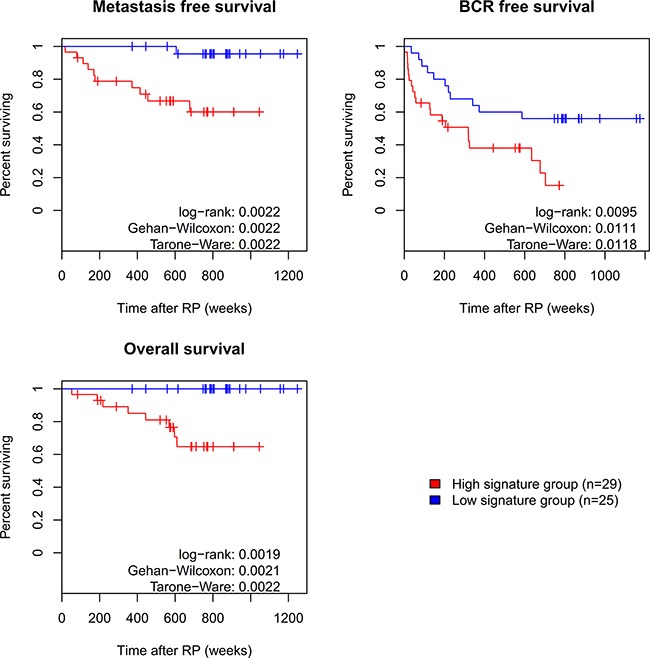
Survival analysis for prognostic PDE4D signature Using the prognostic PDE4D signature to distinguish between outcomes, Kaplan Meier curves for three clinical endpoints were created based on the EMC dataset. Assignment of samples to the high and low signature group was performed by clustering of samples according to their signature values using Partitioning Around Medoids (PAM).

Notably, expression of all long PDE4D isoforms including PDE4D5 and PDE4D7 appears to decrease during PCa progression (see Figure 1 and Supplementary Figures S2-S3), while expression of the super-short PDE4D isoforms PDE4D1 and PDE4D2 seemed to be affected to a lesser extent. On this basis, we decided to create a prognostic signature based on the expression level of PDE4D1/2 relative to the sum of the expression levels of the long PDE4D5, PDE4D7 and PDE4D9 isoforms *PDE*4*D*7. The performance of this signature was then evaluated in the Exon Array cohorts. Since, three datasets had appropriate follow-up available, we used clinical recurrence (CR) defined as development of metastases after RP as clinical endpoint. Overall, our signature performed well in distinguishing patients with CR from those without, yielding AUCs of 0.826, 0.794 and 0.614 for the EMC, Taylor and Erho cohort, respectively (Supplementary Table S4). Since the EMC dataset offered time to biochemical recurrence (BCR), metastases-free as well as overall survival time as follow-up information, we performed a Kaplan-Meier analysis for this dataset using our prognostic PDE4D signature. Two categories (signature high and low) were defined by Partitioning Around Medoids (PAM) and left-censoring was applied, resulting in well separated curves for both metastases-free and overall survival (p < 0.05, see Figure 7). Subsequently, we used Cox proportional hazards regression model to evaluate whether our PDE4D signature is an independent predictor for clinical metastasis, BCR and overall survival, taking into account the pre-operational PSA, Gleason score, pathological stage, surgical margins and patient age. For both metastases-free as well as overall survival, the prognostic PDE4D signature was found to be an independent predictor (p < 0.1), though confidence intervals were large due to low numbers of samples and events (Supplementary Table S6).

## DISCUSSION

Our investigation of the transcriptional dynamics of the *PDE4D* gene locus revealed a previously undescribed promoter switch involving the major contributors of PDE4D activity in normal prostate, namely the PDE4D5 and PDE4D9 long forms, as well as the prostate cancer-associated long isoform PDE4D7 [15, 16]. Unique promoters for each PDE4D isoform, located upstream of the exon(s) encoding their unique N-terminal regions allow for the independent regulation of the different mRNA and corresponding protein expression [6, 9, 33]. Here in this study we provide the first evidence of condition-specific PDE4D promoter switching in a cancer context.

Isoform switching in various genes (such as PKM, CXCR3 and FGR2 [34–36]) during cancer development has been described in several cancer types including prostate cancer [37–40], and likewise tumour-specific isoforms of known genes have been identified previously [41]. Indeed, the androgen receptor variant 7 (AR-V7) provides a particularly important example of a PCa-specific isoform that is constitutively active and ligand-independent, contributing to castration resistance of prostate cancer cells [42, 43]. Furthermore, alternative promoter usage of the androgen-regulated gene TMPRSS2 as part of the TMPRSS2-ERG fusion gene also has been associated with clinical outcome [44, 45].

Interestingly, mounting evidence suggests crosstalk between AR and cAMP signalling pathways, with important cAMP downstream targets such as PKA and ERK interacting either with the AR or AR target genes [4, 46–48]. PDEs, in providing the sole route for degrading cAMP are poised to play a key regulatory role, particularly so as the targeting of particular isoforms to distinct signalling complexes confers a spatial aspect that allows particular isoforms to have specific functional roles [6]. Therefore, it is particularly intriguing to find that specific PDE4D isoforms expressed in prostatic tissues appear to be androgen regulated (PDE4D7 and PDE4D5), suggesting a complex network of interactions that links both pathways. We should, however, mention that studies of PDE4D7 expression in the VCaP prostate cancer cell line implied that it was not directly regulated by AR [15]. However, VCaP harbours genomic rearrangements on chr5q that are characteristic of chromothripsis, and more importantly, PDE4D is reportedly involved in gene fusions with FAM172A and C5orf47 [49]. With regards to the AR-induced up-regulation of PDE4D7 observed in LNCaP cells, these structural rearrangements in VCaP could be involved in a loss of AR-mediated regulation of PDE4D7 due to relocation or deletion of regulatory elements such as AR binding elements. An alternative explanation could be that PDE4D7 expression is indirectly linked to AR activity, as its promoter region overlaps PART1, a known AR target gene that showed clear association with androgen treatment in VCaP [15, 50, 51]. In the Exon Array datasets that we analysed, both genes seem to be co-expressed in prostatic tissues (mean Spearman's rho = 0.7269). However, given the fact that PDE4D5 was significantly down-regulated in LNCaP upon AR stimulation as well, we believe that AR directly influences PDE4D isoform expression through interaction with proximal or distal regulatory elements [52, 53]. This hypothesis is supported by the ChIP-seq data for AR, which identifies numerous binding peaks for AR in the PDE4D gene locus, including the PDE4D5 and PDE4D7/PART1 promoter regions.

Similarly, ERG seems to have a major contribution on PDE4D isoform expression, with us previously reporting that PDE4D7 is up-regulated in TMPRSS2-ERG positive PCa [16]. Here, we provided further ChIP-seq support for ERG involvement in PDE4D expression. However, the Exon Array datasets analysed here do not provide conclusive evidence for a link of ERG overexpression with other isoforms, such as PDE4D5. It appears therefore plausible that ERG overexpression may be specifically linked to PDE4D7 expression, highlighting a connection of the latter to the AR pathway, as well as its potential oncogenic role [15].

To investigate whether DNA methylation could be involved in the promoter switch uncovered in this study, we analysed three independent datasets based on different technologies, whereupon we discovered consistent increases of DNA methylation near the PDE4D5 TSS in PCa samples. In conjunction with the observed AR-mediated down-regulation of PDE4D5, these results could well explain the profound down-regulation of PDE4D5 in localized and advanced PCa and could hint at a protective function in normal prostate that is inhibited by gene silencing in PCa. In addition, we found increased DNA methylation near the PDE4D1/2 TSS that could not be linked to significantly altered gene expression, while other isoforms showing differential methylation (PDE4D4, PDE4D8) do not seem to be consistently expressed in prostatic tissues. Indeed, it is even possible that the increased DNA methylation in the promoter regions of specific PDE4D isoforms might induce promoter switching to PDE4D7 by inhibiting expression of other PDE4D isoforms.

Unlike PDE4D5, PDE4D9 does not show signs of androgen regulation despite being down-regulated in PCa and we could not find evidence for DNA methylation-mediated regulation of PDE4D9 expression in PCa. Thus, its transcriptional regulation in PCa remains unclear at this point and solicits further study.

Taken together, the observed switch in isoform usage might imply that regulatory mechanisms of PDE4D-catalyzed cAMP degradation are subjected to AR signalling in PCa cells that, in turn, indicates that PDE4D7-specific protein domains are necessary to regulate cAMP signalling in an androgen-dependent manner, offering a potentially new drug target [15, 16, 18]. Moreover, with the transition to an androgen-independent state, expression of long PDE4D isoforms seems to fade, reaching its minimum in castration-resistant conditions and distant metastases, while expression of the super-short isoforms PDE4D1 and PDE4D2 appears to remain rather stable. Importantly, these super-short isoforms contain the catalytic domain of PDE4D but lack the UCR1/UCR2 domains seen in long PDE4D isoforms, a module that confers regulation by various kinases and influences intracellular targeting [6, 18].

Hence, this effective loss of regulation of PDE4D activity can be expected to generate profound changes in compartmentalized cAMP signaling due to altered spatial localization and cross-talk governing cAMP degradation, and may thereby contribute to cancer aggressiveness similarly to mechanisms suggested for MAPKs [54] and AR in form of its splice variant AR-V7 [43].

PDE4D isoform composition appears to have merit in being used as a diagnostic signature following the expression of PDE4D7 and PDE4D5, as well as serving as a prognostic signature following the difference between the expression of long and short PDE4D isoforms. Evaluating both signatures, we found that they exhibited good performance in distinguishing PCa from normal tissue and progressive from non-progressive samples, respectively. Importantly, diagnostic performance was robust to differences in technology, data processing, as well as potential differences in composition and patient characteristics of the used cohorts, demonstrating a high cross-platform reproducibility of PDE4D isoforms as PCa biomarker and yielding results comparable to the established PCa-marker PCA3 in all tested cohorts. Hence, with further optimization to an appropriate test platform prior to clinical utilization, we could imagine that such signatures might provide a valuable addition to complement existing test procedures.

When applying our diagnostic signature to prostate biopsies, PDE4D isoform expression appeared to return to its ‘normal’ state with increasing distance from the tumour, whereas the tumour edge showed an intermediate signal. This observation could hint at a ‘field effect’ of the tumour on and/or crosstalk of the tumour cells with the surrounding microenvironment [55–58]. It would therefore be fascinating to further explore in the future whether such a ‘field effect’ indeed influences PDE4D isoform composition, effectively increasing the target area for biopsies, or whether our observations were caused by averaging signals from adjacent tumour and normal cells. If validated, an increased target area could boost accuracy of prostate biopsies, reducing the number of false negative tests. Furthermore, it would be highly interesting to see whether reversing the isoform composition to its normal state has an influence on prostate cell phenotype and behaviour.

While our study focused on PDE4D isoform expression in primary PCa samples, genomic alterations of the PDE4D locus such as microdeletions have been observed in other cancers [27]. Moreover, a recent study found that mutations in other members of the PDE family could be related to PCa by affecting intracellular cAMP and/or cGMP levels [59]. Considering the large number of PDE genes and isoforms as well as the tight regulation of cAMP signalling and its degradation, it is very well possible that PDEs such as PDE4D are key players in other conditions, as the broad panel of associated diseases underscores [10–14]. Therefore, it is worthwhile to extend the presented study and screen the expression profiles of all known PDEs in various tissues and conditions to define basal expression levels and reveal potential alterations and novel targets for drug interventions.

Taken together, our findings highlight the potential of PDE4D isoforms to be promising new biomarkers and potential therapeutic targets for localized and advanced prostate cancer.

## MATERIALS AND METHODS

### Analysis of PDE4D isoform expression in prostate tissues

Quantification of PDE4D isoforms in patient materials was performed by qRT-PCR as described in [16]. In addition, six independent Exon Array datasets were used in this study and raw CEL files were obtained via Gene Expression Omnibus (GEO) or personal communication. The datasets comprised GSE21034 [25], GSE29079 [30], GSE46691 [60], GSE32875 [28] as well as patient samples from GSE41410 [61, 62] and samples published in [63]. These datasets are referred to as ‘Taylor’, ‘Brase’, ‘Erho’, ‘Rajan’, ‘EMC’, and ‘Nijmegen’, respectively.

Of note, patients *PCA0041*, *PCA0042* and *PCA0119* of the Taylor dataset were marked as ‘treated with salvage radical prostatectomy (RP)’, meaning they previously failed radiotherapy treatment and were subsequently treated with RP. Therefore, Exon Array expression data for *PCA0119* were not used for survival analysis.

Raw data were processed and RMA normalized using the aroma.affymetrix R-package ([64], CDF used: HuEx-1_0-stv2,extendedR3,A20071112,EP.CDF, see http://www.aroma-project.org/). Expression of transcript isoforms was measured by using log2-transformed intensity values of isoform-specific probesets: PDE4D1/2 (2858166); PDE4D3 (2858290, 2858291); PDE4D4 (2858368, 2858369, 2858370); PDE4D5 (2858345, 2858346, 2858347); PDE4D6 (2858155, 2858156); PDE4D7 (2858406, 2858407, 2858408); PDE4D8 (2858257, 2858258); PDE4D9 (2858240, 2858241). These intensity values were normalized to a set of reference genes (HPRT1, PUM1, TBP, POLR2A, TUBA1B) by using the mean intensity of ‘core’ probesets of each gene's transcript cluster (3991698, 2404254, 2937984, 3453732, 3708704) to estimate gene expression and then using the average reference gene expression as normalization factor. This normalization factor was subtracted from the probeset intensity values, and normalized probeset expression was subsequently averaged per PDE4D isoform. In addition, expression of the PCa associated genes was normalized the same way as PDE4D, using ‘core’ and ‘extended’ probesets of transcript cluster 3175538 to measure PCA3 as well as 3931765 for ERG and 2811145 for PART1.

Lastly, level 3 processed RNA-seq expression values for PRAD samples were obtained from TCGA (https://tcga-data.nci.nih.gov/) via the TCGA-Assembler R-package [65]. For each sample, the RSEM ‘scaled estimate’ values were used and multiplied by 10^6^ to convert the values to transcripts per million (TPM). Error bars in plots represent standard deviation unless stated otherwise.

### Analysis of deletions of PDE4D and impact on isoform expression

Gene-level copy number alterations were obtained from TCGA via the TCGA-Assembler R-package [65] and a cut-off of ±log2(1.5/2) was used to call gains and losses of genetic material, respectively. A Wilcoxon-Mann-Whitney test was used to identify significant changes in expression of PDE4D isoforms between samples with and without alterations.

### Evaluation of AR and ERG expression / binding on PDE4D transcription

To determine the (TMPRSS2-)ERG status of patient samples in Exon Array cohorts, we used relative ERG expression values and applied Partitioning Around Medoids (PAM, R-package ‘cluster’, k = 2) to assign the patient samples to the ERG positive or negative group based on expression. Lastly, a Wilcoxon-Mann-Whitney-test was used to detect statistically significant differences (p < 0.05) between the ERG positive and ERG negative samples. Likewise, differences between R1881 treated and untreated LNCaP cells [28] were tested using a Wilcoxon-Mann-Whitney-test. To investigate transcription factor binding, public ChIP-seq peaks for AR and ERG were obtained from GEO (GSE14092) and overlapped with PDE4D TSS ± 2 kb regions using bedtools [66] after conversion to hg19 coordinates using the liftOver executable (https://genome.ucsc.edu/cgi-bin/hgLiftOver). Distances of the nearest AR and ERG peaks to each PDE4D isoform TSS were calculated by ‘bedtools closest’ using the options ‘-k 5 and -d’. Data visualization was based on the ggBio R-package [67]. Enrichment of AR and ERG peaks in the PDE4D gene locus was investigated by counting the number of ChIP-seq peaks of each transcription factor within 21,209 RefSeq gene loci (hg19) as well as randomly sampled genomic regions of 1.5 Mb. Unique gene loci were defined by the minimum and maximum chromosomal coordinates of RefSeq NM and NR transcripts belonging to the same gene identifier after associating them to HGNC gene symbols using biomaRt [68] and excluding minor chromosomes and haplotypes. For each chromosome, random regions were sampled according to: *number of regions* = (chromosome size in Mb * 1000) and any regions overlapping the PDE4D gene locus were excluded. Counting was performed by bedtools [66] *annotate* using the option ‘-counts’ and empirical cumulative distribution functions for both transcription factors were created by using the ecdf() function of R-package stats. Hexbinplots were generated using the BoutrosLab.plotting.general R-package (http://labs.oicr.on.ca/boutros-lab/software/bpg).

### Investigation of PDE4D promoter methylation

Public methylation data were downloaded from GEO and TCGA data portal and comprised three different technologies. 1) Deduplicated and extended MeDIP-seq reads (200 nt) deposited under accession number GSE35342 [31] were downloaded from Gene Expression Omnibus (GEO) and processed via the MEDIPS R-package [69]. Using genomic bins of 100 nt for chromosome 5, reads were counted for every sample and differential methylation status of each bin was tested using the following MEDIPS settings as suggested by the authors upon request: ‘diff.method = “edgeR”, prob.method = “poisson”, MeDIP = F, CNV = F’. Bins covering the genomic region of PDE4D including 50 kb flanks and with a Bonferroni-adjusted p-value below 0.01 were selected and merged into larger regions of interest (ROIs) if they were directly adjacent. 2) Pre-processed public bisulfite sequencing (BiS-seq) data available from GEO (GSE41701, [70]) were downloaded, and measured positions found in the genomic region of PDE4D including 50 kb flanks were extracted. For each position, the percentage of reads indicating methylation was calculated by #base calls C / (#base calls C + #base calls T) based on the number of reads covering a particular base. Next, the limma R-package [71, 72] was used to identify positions with significant differences in methylation between PCa vs. benign, as well as CRPC vs. PCa. Positions with FDR < 0.05 were selected and merged into larger regions if they were within 100 nts of each other. 3) TCGA level 3 data for Illumina Infinium HumanMethylation450 BeadChips were downloaded from TCGA data portal and only patients with available clinical information were used for further analysis. Pre-calculated beta values for chromosome 5 were imported into Minfi [73] and annotated using ‘ilmn12.hg19’. Analysis of differential methylation was performed via bumphunter using 100 permutations and ‘cutoff=0.15’. Lastly, any significant probes located within the genomic region of PDE4D including 50 kb flanks were extracted and methylation profiles were correlated to RNA expression via Spearman's correlation coefficient. Visualisation of methylated regions was performed using ggBio [67].

### Analysis of signature performance, survival and independent predictor variable

We created a diagnostic signature based on PDE4D7 expression relative to PDE4D5 expression (PDE4D7-PDE4D5) as well as a prognostic signature for the Exon Array cohorts based on PDE4D1/2 relative to PDE4D5, PDE4D7 and PDE4D9 ((PDE4D1/2) - (PDE4D5+PDE4D7+PDE4D9)). Subsequently, the R-packages ‘ROC’ and ‘survival’ were used to carry out ROC analyses and perform a Cox regression as well as Kaplan-Meier analysis based on available survival data of the EMC dataset [61, 62].

### Quantification of diagnostic PDE4D signature in prostate biopsies

Several biopsy punches (approximately 1 x 2 mm) were taken in a representative tumour area after surgical prostate resections in eighteen different men with prostate cancer. Experimental protocols were approved by the Erasmus MC Medical Ethics Committee following the Medical Research Involving Human Subjects Act. For each patient, these punches were performed within the tumour, at the edge of the tumour area, at 5 and at 10 mm distance to the tumour region. RNA was extracted and qRT-PCRs (quantitative real-time PCR) for PDE4D5 and PDE4D7 were performed as described in [16], using ACTB, HPRT1, TUBA1B, POLR2A, PUM1 and TBP as reference genes. The expression of PDE4D5 and PDE4D7 in each biopsy tissue was normalized as follows: mean(Ct(reference genes)) – Ct(PDE4DX). For each of the eighteen different patients, the normalized expression of PDE4D transcripts within the tumour was set to 1 and expression values for biopsies taken at various distances from the tumour were calculated relative to the expression in the tumour. Lastly, average relative expression and standard error of the mean of PDE4D transcript expression were plotted for each of the respective biopsy locations.

## SUPPLEMENTARY FIGURES AND TABLES














