# Supplementary material for: Human PDE4D isoform composition is deregulated in primary prostate cancer and indicative for disease progression and development of distant metastases
Source: Oncotarget. 2016 Sep 23;7(43):70669–84. doi: 10.18632/oncotarget.12204 (PMC5342582; doi:10.18632/oncotarget.12204)
Supplement: Supplementary file 1 [file oncotarget-07-70669-s001.pdf]

Human PDE4D isoform composition is deregulated in primary prostate cancer and indicative for disease progression and development of distant metastases

SUPPLEMENTARY FIGURES AND TABLES

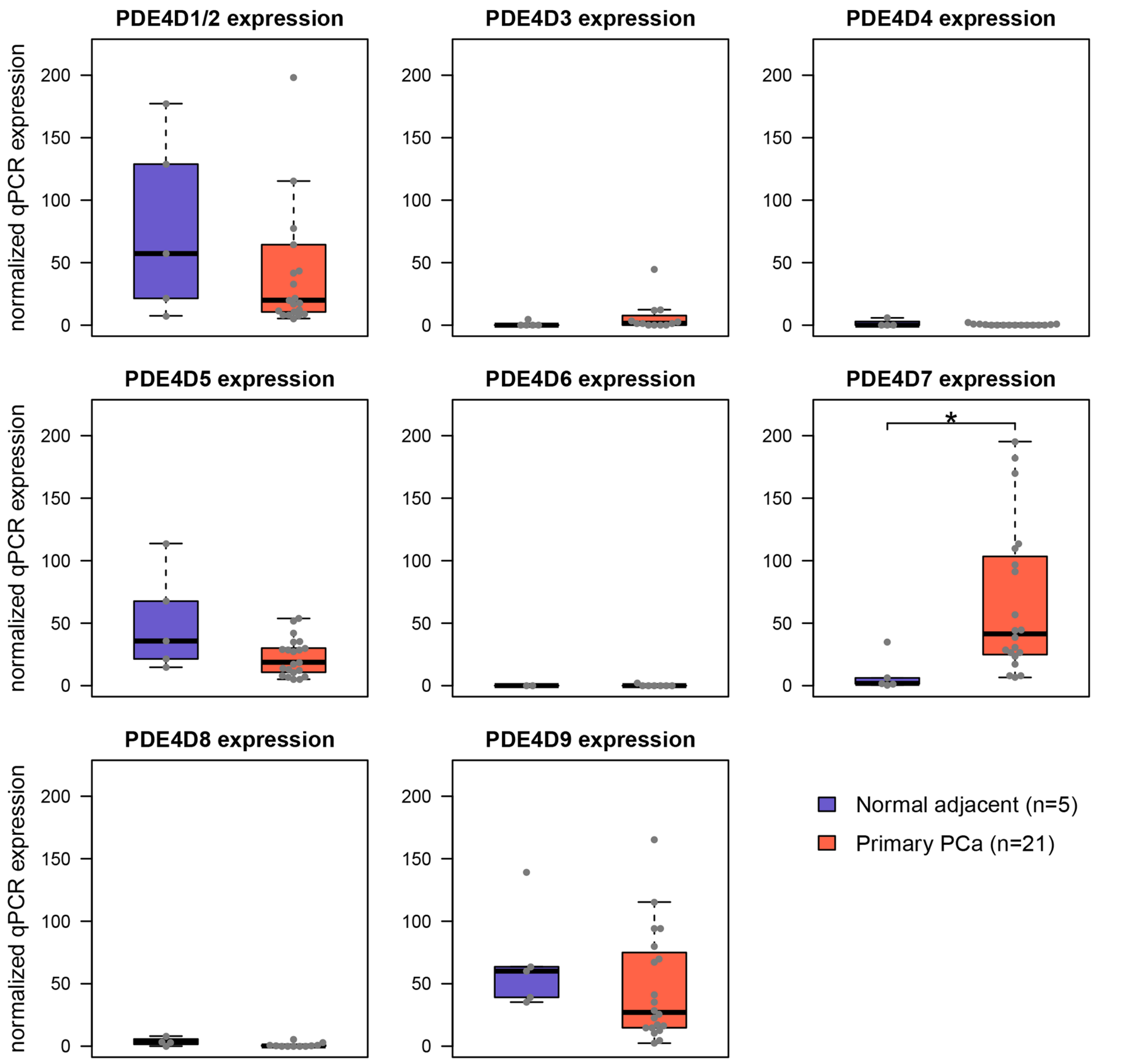

Supplementary Figure S1: Normalized PDE4D isoform expression measured using qRT-PCR in an independent patient cohort.

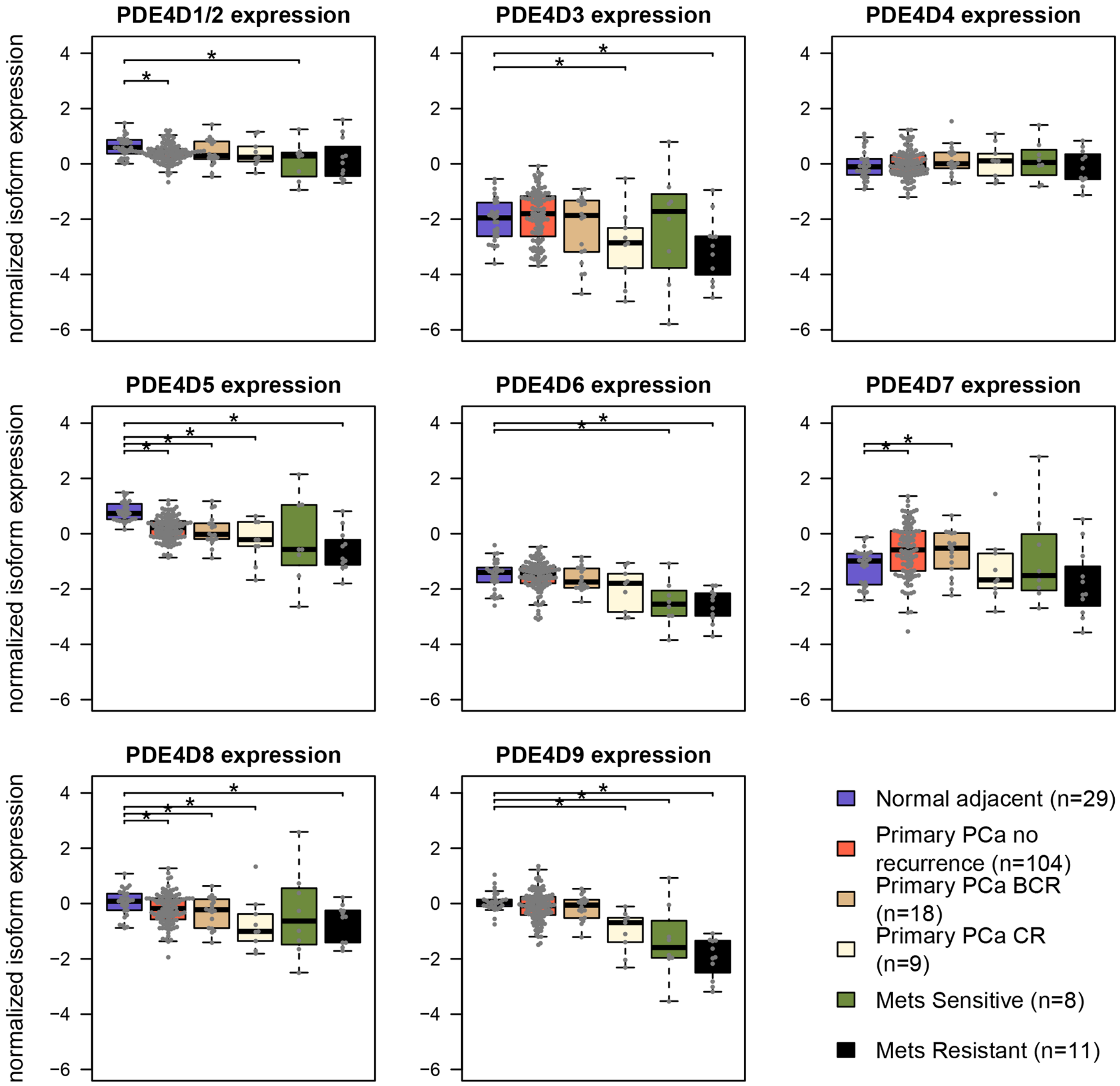

Supplementary Figure S2: Normalized PDE4D isoform expression in the 'Taylor' dataset across different prostate conditions. CR – clinical recurrence, BCR – biochemical recurrence, Mets sensitive – hormone responsive clinical metastasis, Mets resistant – castration resistant clinical metastasis.

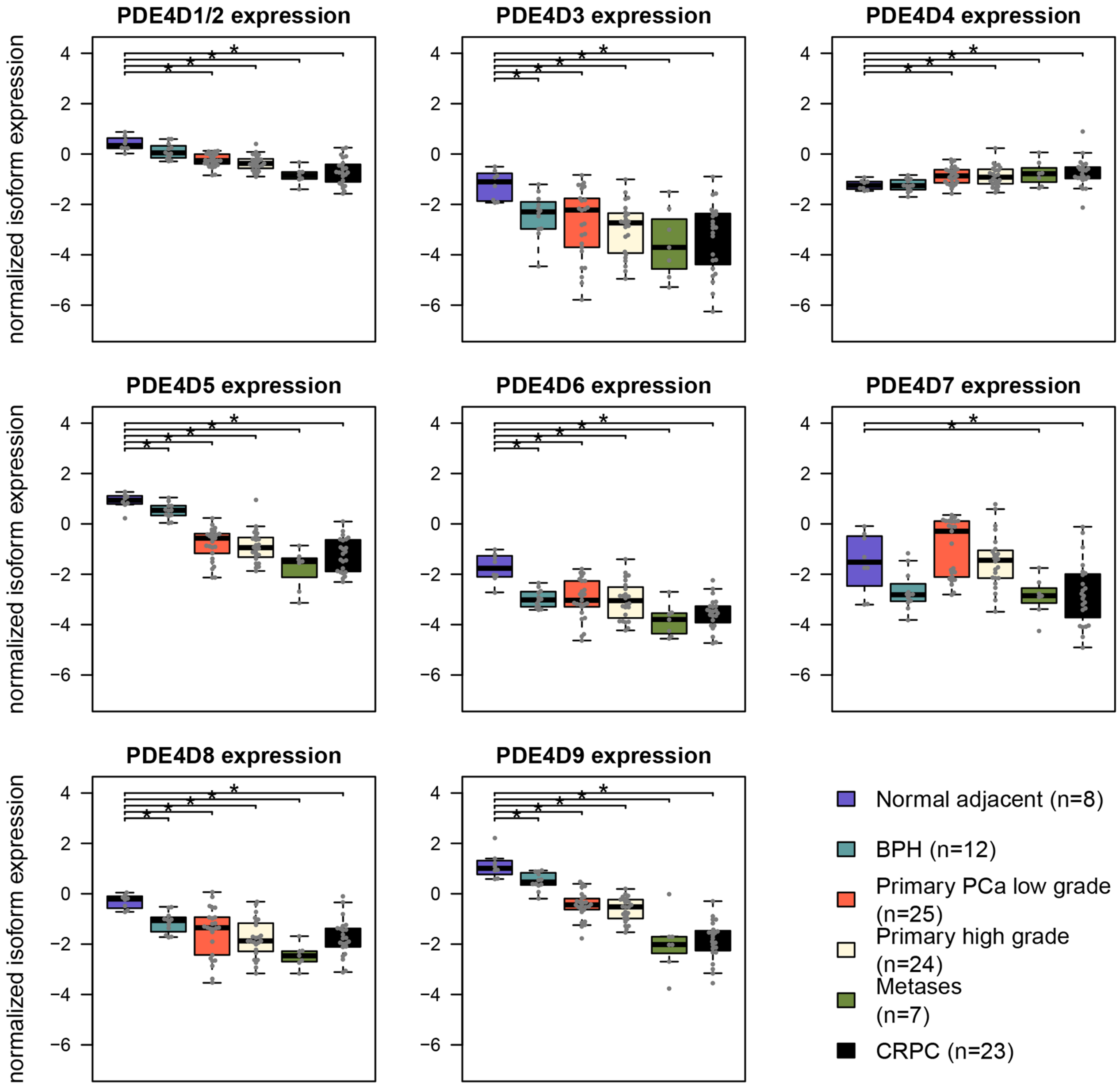

Supplementary Figure S3: Normalized PDE4D isoform expression in the 'Nijmegen' dataset across different prostate conditions. Low grade – Gleason score <= 6, High grade – Gleason score >= 7 recurrence, CPRC – castration resistant prostate cancer.

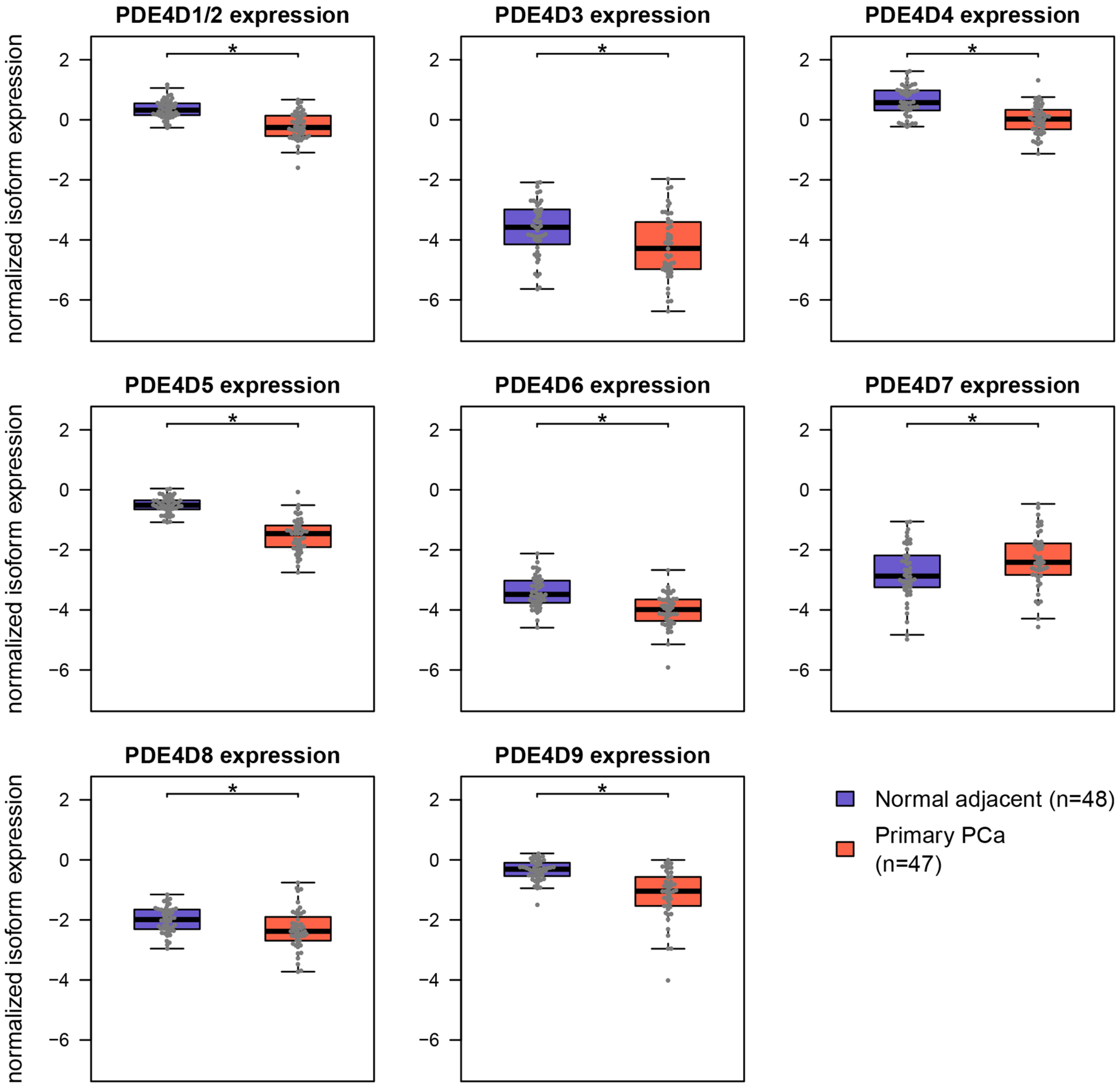

Supplementary Figure S4: Normalized PDE4D isoform expression in the 'Brase' dataset across different prostate conditions.

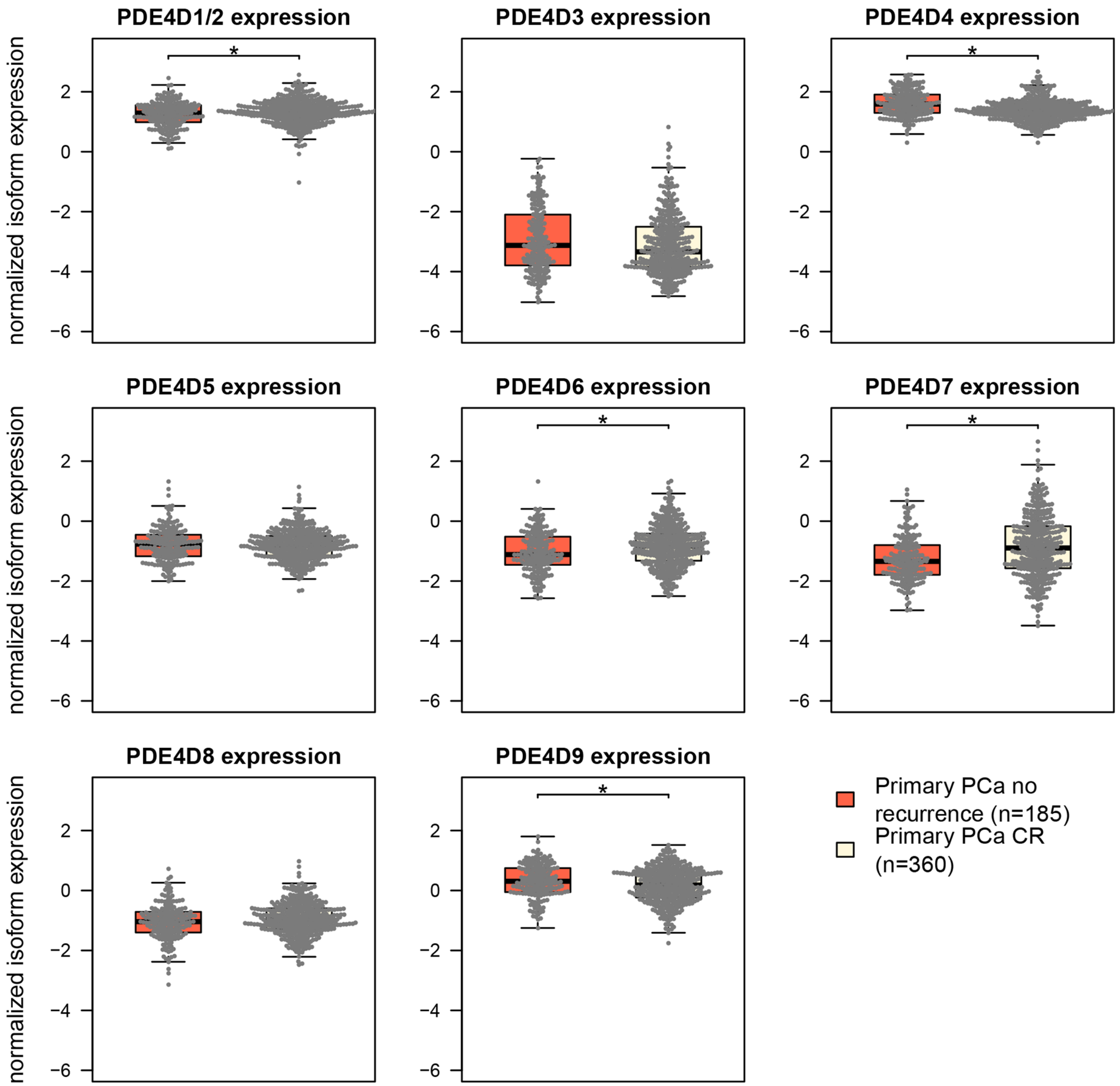

Supplementary Figure S5: Normalized PDE4D isoform expression in the 'Erho dataset across different prostate conditions. CR – clinical recurrence.

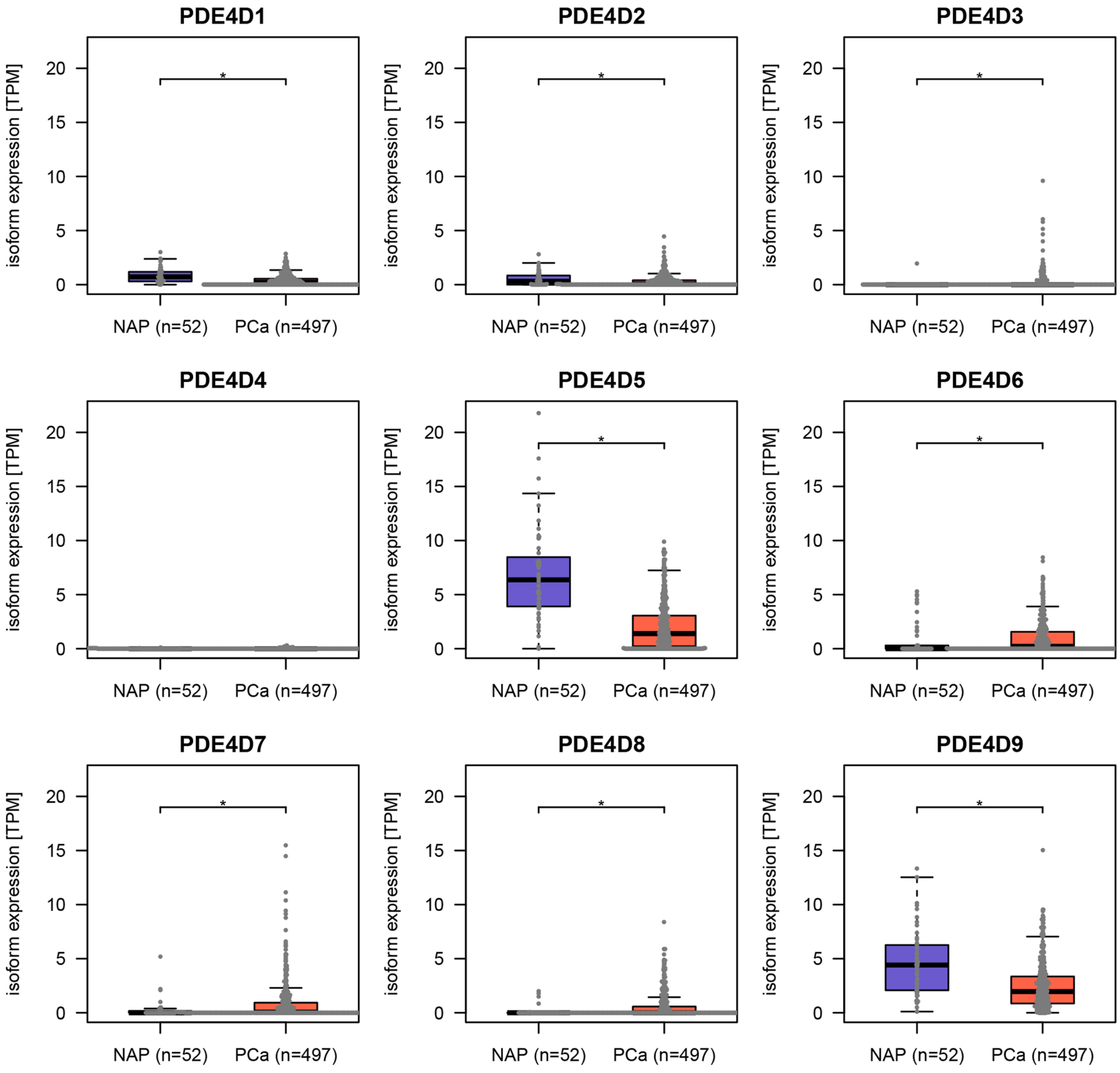

Supplementary Figure S6: PDE4D isoform expression in the TCGA PRAD RNA-seq cohort across different prostate conditions. TPM – transcripts per million. RSEM 'scaled estimates' (TCGA level 3) were converted to TPM (transcripts per million) by multiplying each value by 10^6 and a Wilcoxon-Mann-Whitney test was used to find statistically significant differences in isoform expression.

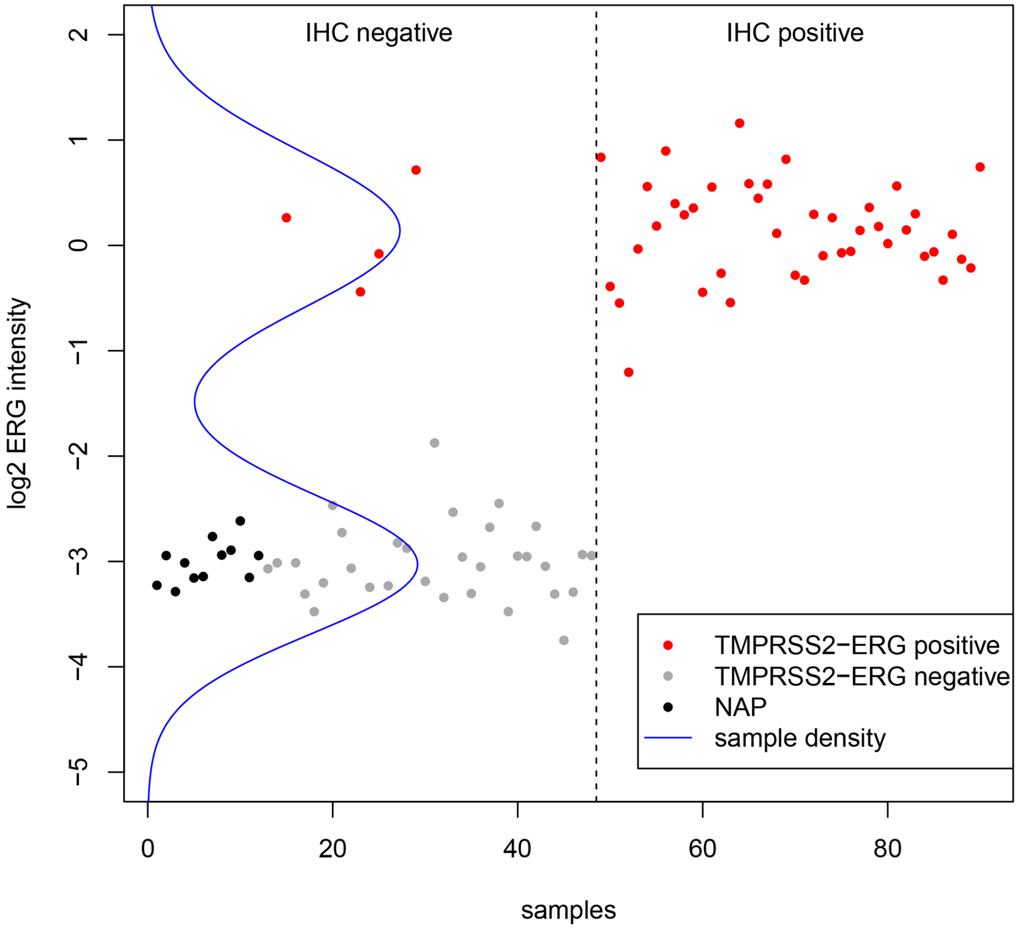

Supplementary Figure S7: Normalized ERG expression in the 'EMC' dataset compared to immunohistochemistry (IHC) results. Samples were clustered based on ERG expression using Partitioning Around Medoids.

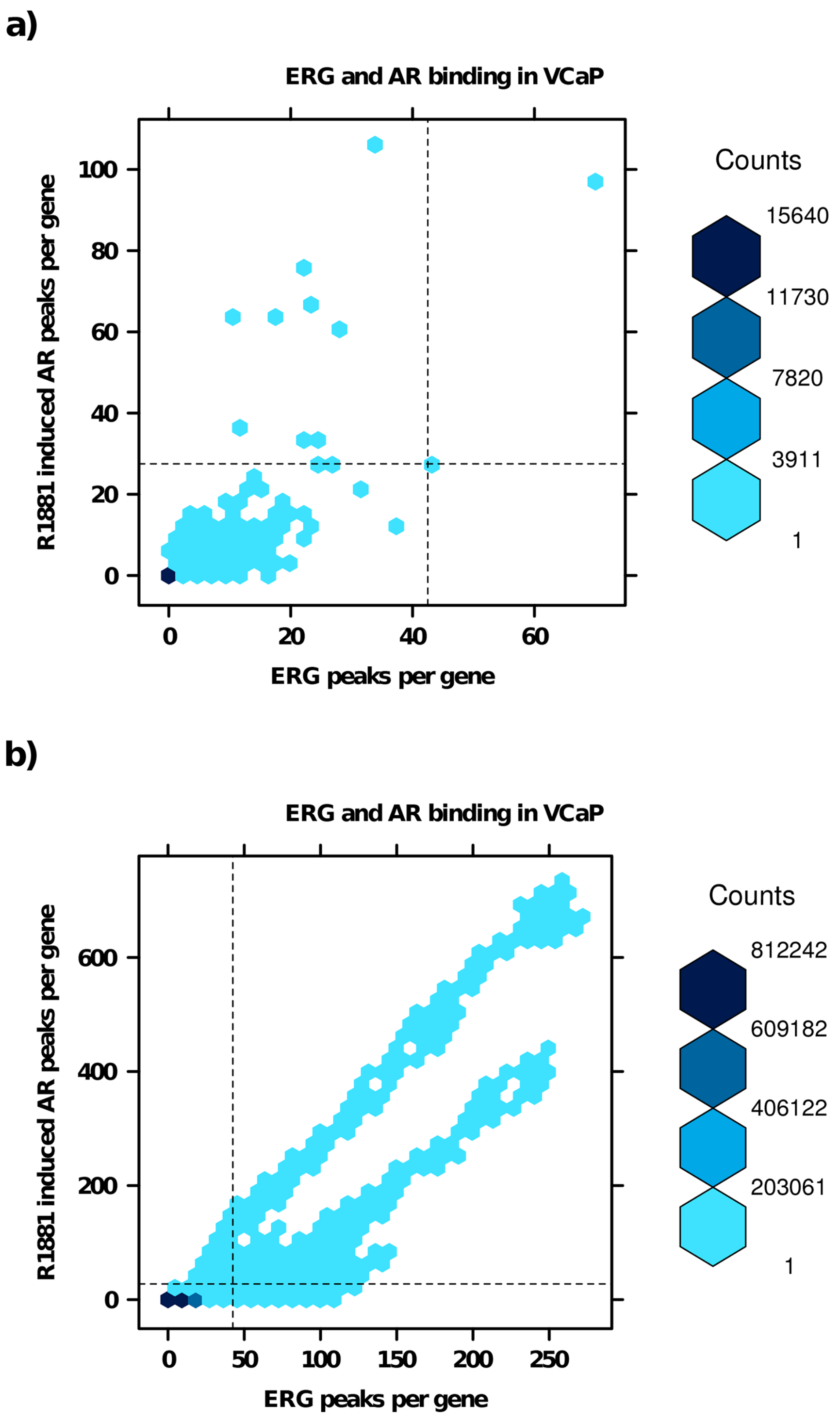

Supplementary Figure S8: Hexbinplot of the number of AR and ERG ChIP-seq peaks per gene and genomic region. Each hexagon represents a number of genes with x ERG and y AR ChIP-seq peaks. Ranges of 'Counts' (number of genes with the same coordinates) are denoted by different colours to represent the density of genes at specific xy-coordinates. **A.** Binding peaks were counted for 21,209 RefSeq gene loci; dotted lines indicate the location of PDE4D relative to the rest of the genome. **B.** Likewise, peaks were counted for randomly selected genomic regions of comparable size to PDE4D (1.5 Mb). Most genes and regions show little to no AR or ERG binding peaks as evident by dark coloured hexagons near (0,0).

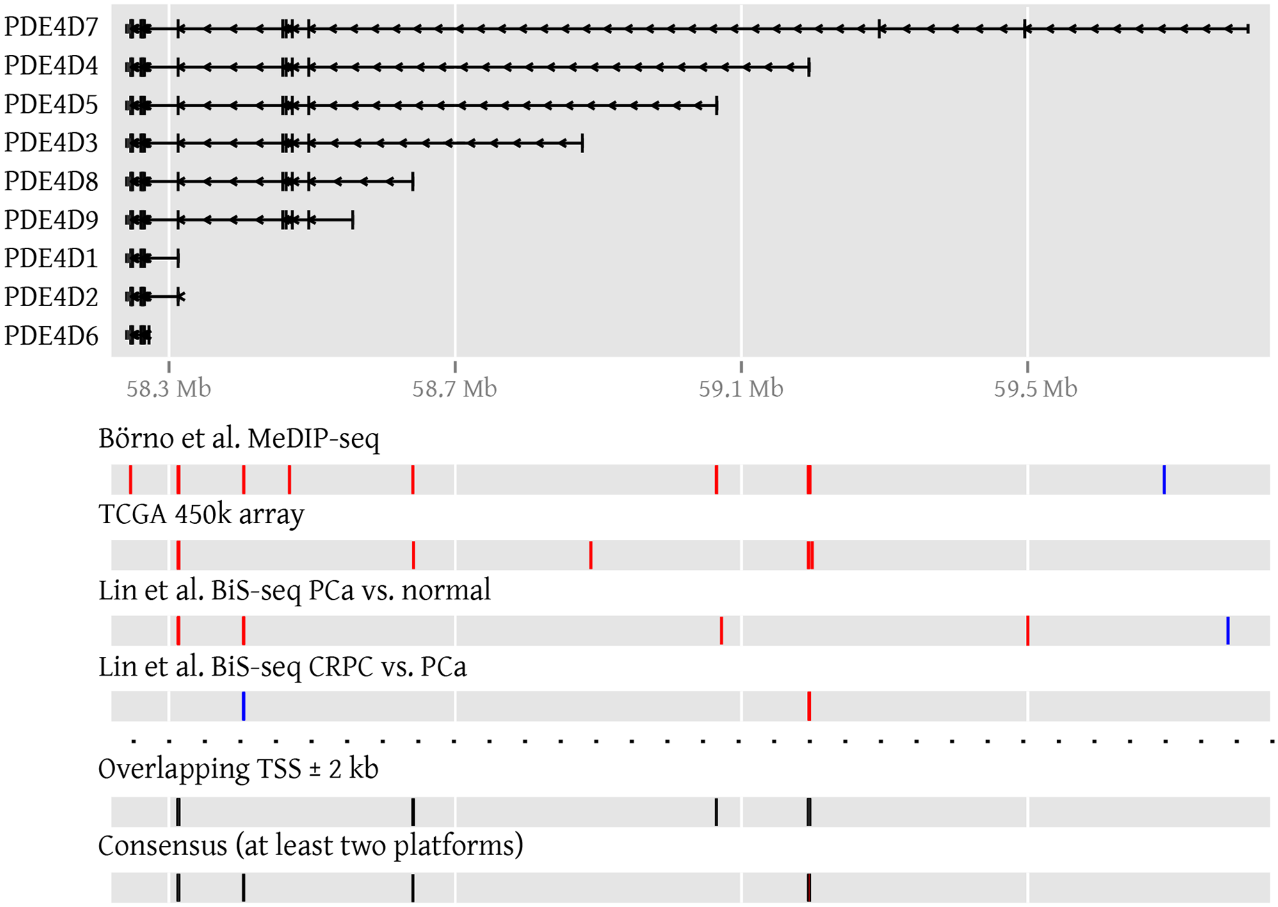

Supplementary Figure S9: Differentially methylated regions identified in several public datasets (Börno et al. - GSE35342, Lin et al. - GSE41701). Hyper-methylated sites are coloured red, while hypo-methylated sites are coloured red. TSS – transcription start site, CPRC – castration resistant prostate cancer.

Supplementary Table S1: Genomic positions of AR ChIP-seq peaks in the VCaP cell line as well as their relative distances to the nine canonical PDE4D transcription start sites.

See Supplementary File 1

Supplementary Table S2: Genomic positions of ERG ChIP-seq peaks in the VCaP cell line as well as their relative distances to the nine canonical PDE4D transcription start sites.

See Supplementary File 2

Supplementary Table S3: Spearman's correlation coefficients between RNA expression of individual PDE4D isoforms and significantly differentially methylated regions (DMRs) located in the PDE4D locus. Correlation coefficients for DMRs in close proximity to a transcription start site (TSS) are marked in bold. PDE4D1 and PDE4D2 expression could not be independently measured and were therefore merged. For completeness, hg19-based coordinates for both TSS have been included.

See Supplementary File 3

Supplementary Table S4: Summary of ROC analyses results for PDE4D signatures. Area under curve (AUC) estimates for the diagnostic and prognostic PDE4D signatures compared to PCA3 in ROC analyses across various cohorts.

See Supplementary File 4

Supplementary Table S5: Pathological Gleason score and pre-operative PSA for the patient samples used to simulate prostate biopsies (NA if unavailable).

See Supplementary File 5

Supplementary Table S6: Summary of univariante and multivariate Cox regression results for PDE4D. Results of Cox regression analysis for three clinical endpoints based on the 'EMC' dataset and using the prognostic PDE4D signature.

See Supplementary File 6
